# Metabolic Dysfunction in Idiopathic Intracranial Hypertension: Current Theories and Updates

**DOI:** 10.14336/AD.2024.1147

**Published:** 2025-11-25

**Authors:** Hui Li, Lu Liu, Yifan Zhou, Huimin Jiang, Weiyue Zhang, Chenxia Zhou, Chuanjie Wu, Chen Zhou, Xunming Ji

**Affiliations:** ^1^Department of Neurology, Xuanwu Hospital, Capital Medical University, Beijing, China.; ^2^Department of Neurosurgery, Xuanwu Hospital, Capital Medical University, Beijing, China.; ^3^Beijing Institute of Brain Disorders, Laboratory of Brain Disorders, Ministry of Science and Technology, Collaborative Innovation Center for Brain Disorders, Beijing Advanced Innovation Center for Big Data-based Precision Medicine, Capital Medical University, Beijing, China.; ^4^Beijing Advanced Innovation Center for Big Data-Based Precision Medicine, School of Biological Science and Medical Engineering, Beihang University, Beijing, China.

**Keywords:** Idiopathic intracranial hypertension, metabolic disease, cerebral venous sinus stenosis, raised intracranial pressure

## Abstract

Idiopathic intracranial hypertension (IIH) is a disease characterized by increased intracranial pressure (ICP) without identifiable secondary causes. While the increased ICP is a critical diagnostic feature, the underlying pathophysiological mechanisms remain unclear. Previous theories have suggested cerebrospinal fluid (CSF) overproduction, impaired reabsorption, or circulatory obstruction as potential causes. Emerging evidence indicates that IIH may not be solely a central nervous system disorder but also a systemic metabolic disorder. Metabolic and hormonal dysregulation features, including hyperleptinemia, adipocyte leptin hypersecretion, increased insulin resistance, and androgen excess, have been noted in IIH. Furthermore, the targeted blockade of the cortisol-producing enzyme 11β-hydroxysteroid dehydrogenase type 1 has demonstrated therapeutic potential in treating IIH. Consequently, the role of metabolic dysfunction and hormonal imbalance in IIH warrants consideration. This review aims to provide a comprehensive update on current theories regarding the mechanisms of metabolic dysfunction in IIH.

## 1. Introduction

Idiopathic intracranial hypertension (IIH), also referred to as pseudotumor cerebri or benign intracranial hypertension, is a disorder characterized by elevated intracranial pressure (ICP) without an identifiable underlying cause [1, 2]. The incidence is highest in young, obese women of childbearing age, with a prevalence four times that of men [3-6]. Given the strong correlation between body mass index (BMI) and IIH, the incidence and prevalence of IIH have more than doubled over the past two decades [7]. This trend is anticipated to persist as the global burden of obesity continues to rise. Furthermore, IIH imposes a significant economic burden due to the associated morbidity, which results in lost income and escalating healthcare costs that have increased fivefold over the past decade [3, 8].

The common symptoms of IIH include headaches, pulsatile tinnitus, transient blurred vision, diplopia, vision loss, and cognitive impairment. Among these, headaches and vision impairment are the most common symptoms [9-12]. Due to the atypical symptoms of IIH and the unclear etiology of elevated ICP, a diagnosis of IIH diagnosis requires direct evidence of papilledema, elevated ICP (≥250 mmH_2_O), normal cerebrospinal fluid (CSF) composition, and no structural causes of elevated ICP on computed tomography (CT) or magnetic resonance imaging (MRI) [2]. The primary objectives of IIH treatment are to address the underlying causes of increased ICP, to safeguard vision, and to provide effective management of headache episodes. In line with these principles, the guidelines advocate weight management as the primary approach, followed by carbonic anhydrase inhibitors such as acetazolamide, which lower ICP by inhibiting CSF production and further relieving the symptoms of elevated ICP [13-15]. For patients with refractory or fulminant IIH, invasive interventions such as venous sinus stenting (VSS), CSF shunting, bariatric surgery and optic nerve sheath decompression are often considered [15]. However, a 2015 Cochrane review concluded that insufficient evidence exists to determine which treatments may benefit IIH [16]. Thus, there is an unmet medical need for IIH-specific diagnostic indicators and more effective targeted therapies, highlighting the importance of further understanding the pathophysiology of IIH.

Elevated ICP is believed to result from disturbances in CSF homeostasis. Previous studies have suggested that IIH may be related to increased CSF production by the choroid plexus, impaired reabsorption of arachnoid granulations (AGs) in the cortical venous sinuses, or venous hypertension secondary to stenotic structural abnormalities [17, 18]. Additionally, the lymphatic system, comprising perivascular spaces (PVS) surrounding the cerebral veins and arteries, facilitates peri-arterial solute exchange before waste products and excess interstitial fluid (ISF) through the veins [19]. Increasing evidence suggests that impaired lymphatic drainage contributes to the pathogenesis of IIH [20-26]. However, the exact pathogenesis of IIH remains unclear, and an explanation or molecular mechanism for a single homeostatic disturbance has not been found [1, 15].

Awareness of IIH is increasing, and it is now recognized as more than a central nervous system (CNS) disorder, with growing evidence of systemic metabolic disturbances beyond those caused by obesity. For instance, systemic hormonal dysregulation, including a distinct profile of androgen excess, has been observed in IIH [27]. Patients with IIH also show increased susceptibility to insulin resistance in the presence of adipocyte leptin overproduction and hyperleptinemia [28]. Additionally, a recent metabolomics study using nuclear magnetic resonance spectroscopy identified significant metabolic abnormalities in the urine, serum and CSF of the participants suffering from IIH, indicating generalized metabolic dysregulation [29]. Metabolically targeted therapies, such as inhibiting the adrenocorticotropic hormone-producing enzyme 11β-hydroxysteroid dehydrogenase type 1 (11β-HSD1), have shown potential in treating IIH [30]. Glucagon-like peptide-1 (GLP-1), which is found in the choroid plexus, may help treat IIH by reducing CSF production [31, 32]. Studies have shown that IIH patients treated with the GLP-1 receptor agonist exendin-4 experienced a rapid and sustained decrease in ICP and improvement in clinical symptoms, even without significant weight loss [32]. Given the emerging evidence, it is likely that IIH is triggered by systemic metabolic derangements that ultimately lead to disturbances in CSF dynamics. This review aims to provide a comprehensive update on current theories regarding the mechanisms of metabolic dysfunction in IIH.

## 2. Pathophysiology of IIH

**Figure 1.**
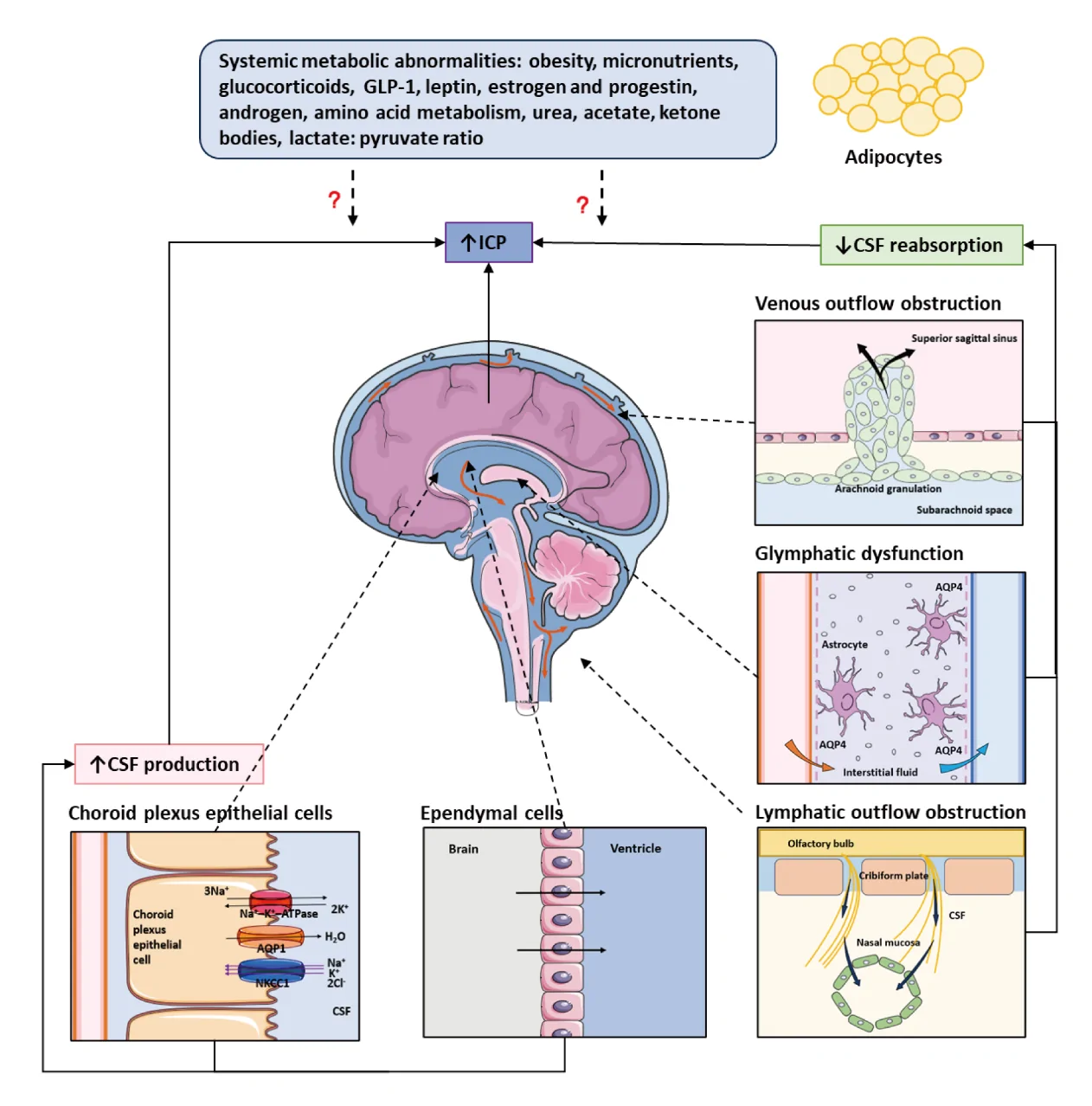
**Schematic representation of the potential pathophysiological mechanisms underlying IIH**. CSF is primarily produced by the epithelial cells of the choroid plexus, with a small portion produced by epithelial cells of the ventricular system. Alterations in CSF production are thought to involve upregulation of Na^+^/K^+^ ATPase transporter activity or dysfunction of AQP1 channel, both located on the apical surface of choroid plexus epithelial cells. The role of the NKCC1 cotransporter, also expressed on the apical membrane of these cells, remains controversial. Impaired CSF reabsorption or drainage may be attributed to factors such as obstruction of arachnoid granulations, venous drainage obstruction, reduced perineural CSF drainage rates, and lymphatic dysfunction, all of which contribute to reduced CSF clearance and increased ICP. Systemic metabolic abnormalities, including obesity, can further elevate ICP by influencing CSF production and reabsorption process. Abbreviations: IIH, idiopathic intracranial hypertension; CSF: Cerebrospinal fluid; AQP1, aquaporin 1; NKCC1, Na^+^-K^+^-2Cl^-^ cotransporter; ICP, intracranial pressure.

The dysregulation of CSF dynamics is thought to play a significant role in the pathophysiology of IIH, which may involve overproduction of CSF by the choroid plexus, reduced reabsorption via AGs, venous outflow obstruction, and overflow of the glymphatic system (Fig. 1). CSF is primarily produced by the choroid plexus within the lateral ventricles and flows sequentially through the third and fourth ventricles to reach the subarachnoid space. It then drains through the AGs into the cortical venous sinuses, driven by a pressure gradient mechanism [33]. Although the exact mechanism of CSF circulation remains incompletely understood, both venous and lymphatic outflow pathways are known to contribute [1, 21].

### 2.1 CSF Production

Under physiological conditions, the choroid plexus is the primary site of CSF production, accounting for approximately 80% of the total CSF [34-36]. Epithelial structures of the choroid plexus regulate the composition of CSF through selectively controlling the movement of solute and water. Ion translocation and subsequent osmotic gradient formation facilitate water entry into the ventricles via aquaporin-1 (AQP1) channels on the apical surface of choroid plexus cells [37, 38].

Changes in osmotic pressure affect CSF production by the choroid plexus, with pharmacological studies demonstrating the role of ion transporter inhibition in regulating CSF secretion [39-41]. At the basolateral membrane, the Na^+^-HCO_3_^-^ cotransporter (NBC) utilizes ionic gradient to promote HCO_3_^-^ accumulation in the epithelium. Intracellular carbonic anhydrase further promotes this ionic accumulation by catalyzing the production of HCO_3_^-^ and H^+^ from H_2_O and CO_2_ [42]. Currently, anhydrase inhibitors such as acetazolamide, which reduces ICP by inhibiting CSF production, are critical therapeutic modalities for IIH [15, 16]. In parallel with the activities at the basolateral membrane, transporters at the apical membrane involve the action of the Na^+^-K^+^-ATPase pump and inwardly rectifying anionic currents. And direct inhibition of the Na^+^-K^+^-ATPase transporter on the apical surface can reduce CSF production by up to 80%. The role of Na^+^-K^+^-2Cl^-^ cotransporter (NKCC1) expressed on the apical membrane of the choroid plexus, remains controversial. Studies have shown that the NKCC1 inhibitor bumetanide reduces CSF production in choroid plexus epithelial cells [43-46]. One study suggested that bumetanide, a drug acting on NKCC1 channels, partially inhibits AQP1 at high concentrations [38, 47]. Recent studies have identified a variety of metabolic abnormalities, including obesity, micronutrients, leptin, estrogen and progesterone, androgens, etc., which may increase CSF production by upregulating ion transporters and AQP1 activity, representing potential pathophysiological mechanisms and therapeutic targets for IIH [11, 48-54].

At present, it is controversial whether increased CSF production is a pathological mechanism of IIH. An early study suggested that increased CSF production could contribute to the development of IIH [55]. However, subsequent studies found no difference in CSF production or the size of the choroid plexus between IIH patients and healthy volunteers [56, 57]. In addition, patients with choroid plexus papilloma, a neuroepithelial tumor characterized by increased CSF production, develop enlarged ventricles and hydrocephalus, whereas these findings were not seen in IIH [18, 58]. Therefore, further investigation of how metabolic abnormalities regulate ion transporters and AQP1 activity is needed to clarify the relationship between CSF production and IIH, providing insights for IIH management.

### 2.2. CSF Circulation

#### 2.2.1 CSF Reabsorption-AGs

As a channel for CSF from the subarachnoid space to the cerebral venous sinuses, AGs and arachnoid villi play a vital role in maintaining CSF homeostasis and ICP regulation. Recent studies have demonstrated significant heterogeneity in AG size, frequency, and distribution, showing that ICP can be restored to normal despite the physical absence of AGs [59].

The role of AGs in IIH may be either causal or compensatory. Cerebral venous sinus stenosis (CVSS), especially localized stenosis at the junction of the transverse and sigmoid sinuses, occurs in up to 90% of patients with IIH [60]. Similarly, AGs are frequently found near the transverse, sigmoid, and sagittal sinuses, where they may enlarge and protrude into the lumen of the dural sinus, potentially causing local stenosis [61, 62]. AGs may also serve as a compensatory function in IIH patients [62-64]. Their number and size tend to increase with rising subarachnoid CSF pressure [65]. In a retrospective chart review, Watane et al. found that an increase in AGs was associated with a reduction in IIH signs[62-64]. Therefore, it remains controversial whether the increase in AGs serves as a compensatory mechanism to facilitate CSF outflow and reduce ICP, or if the ICP rise results from sinus herniation and subsequent stenosis [66]. In addition, various types of AGs may play distinct roles in CSF reabsorption. Pathological and radiological studies have identified a specific type of AG, termed 'vascular AG' [67]. Similarly to the lymphatic outflow pathway, CSF drainage from the subarachnoid space via 'non-vascular granules' may regulate ICP, while CSF drainage through 'vascular granules' in the lymphatic system may aid in clearing cerebral metabolites. Thus, impaired clearance of cerebral metabolites due to vascular AG dysfunction may contribute to IIH symptoms, offering a new perspective on the condition's pathological mechanisms.

#### 2.2.2 CSF Drainage Pathway-Venous System

As a significant predisposing factor for IIH symptoms, CVSS impedes efficient venous outflow pathway performance for lymphatic reabsorption and direct reabsorption aimed at balancing ICP [20, 68-70]. Moreover, cerebral venous malformations or sinus thrombosis can increase cerebral venous pressure, culminating in increased ICP [71-73].CVSS has been reported to be present in up to 90% of IIH patients, but the relationship between CVSS and increased ICP remains controversial [74]. First, CVSS can reduce the ICP-venous pressure gradient by raising cerebral venous pressure, thereby limiting CSF reabsorption [70, 75]. Elevated ICP further compresses the cerebral venous sinuses and exacerbates the CVSS, perpetuating this vicious cycle [70]. Finally, through a feedback mechanism, ICP and venous pressure progressively increase until reaching a state of high-pressure equilibrium [76]. However, further evidence suggests that high ICP in IIH is a risk factor for CVSS. This contention is indirectly supported by recurrent stenosis post-VSS in patients with IIH [77]. There is a tendency for ICP to be consistently high in patients with IIH, and VSS is only a focal disruption of the positive feedback loop. It does not address the underlying stimulatory mechanisms leading to diffuse CVSS, which explains the natural tendency for stent failure [77]. The immediate relief of stenosis after lumbar puncture or CSF drainage suggests that CVSS may also be a secondary consequence of elevated ICP [78, 79]. Moreover, some patients with CVSS do not have elevated ICP upon examination, and the degree of stenosis has no relation to CSF opening pressure or clinical symptoms [60]. Therefore, it remains uncertain whether CVSS is the primary cause of elevated ICP or a secondary consequence of external compression of the vessel due to elevated ICP.

Currently, an increasing amount of evidence supports the use of VSS as a viable option for medically refractory IIH [80]. The study by Lenck et al. demonstrated that performing VSS effectively alleviates IIH symptoms regardless of the type of CVSS [69]. While ventriculoperitoneal shunt (VPS) or lumbar spinal portosystemic shunt (LPS) has a long history of use in IIH treatment, VSS as an etiological treatment is theoretically more appropriate for IIH induced by CVSS. However, there is no high-quality evidence-based medical data on its efficacy and safety. A variety of metabolic abnormalities, including obesity, glucocorticoids, and GLP-1, have been identified in patients with IIH, which may cause elevation of ICP by increasing central venous pressure (CVP) and obstructing cerebral venous outflow from the CSF [28, 32, 81]. Exploring the effects and mechanisms of various metabolic abnormalities on ICP will help to clarify the relationship between CVSS and IIH and provide a new discriminative index and pharmacological target for the treatment of IIH accompanied by CVSS and CVSS-induced IIH.

#### 2.2.3 CSF Drainage Pathway-Lymphatic System

The lymphatic drainage pathway offers an alternative mechanism for CSF reabsorption, contributing to 14%~47% of total CSF drainage [23, 82-86]. In 2012, J. Iliff et al. first demonstrated that the astrocyte-mediated cerebral PVS is a drainage pathway for CSF-ISF, termed the glial lymphatic system [19]. Additionally, lymphatic vessels constitute a parallel circulatory system to blood vessels [87, 88]. The lymphatic outflow pathway may act as a compensatory mechanism when the venous efflux pathway is compromised, potentially explaining why some patients with impaired CVSS or extracranial venous outflow do not exhibit elevated ICP [89]. Evidence indicates that lymphatic dysfunction may occur in patients with IIH [23]. A volumetric MRI imaging study observed increased interstitial volume and CSF accumulation in the subarachnoid space and PVS of IIH patients, suggesting an imbalance in CSF circulation [25, 90]. The most common radiological manifestations include optic nerve sheath distension and optic nerve tortuosity, as well as Meckel's cave enlargement and Dorello's canal enlargement [91-93].

Additionally, metabolic abnormalities, including obesity, leptin, estrogen, and progesterone, may contribute to IIH by impairing lymphatic CSF drainage. The lymphatic system clears ISF along with proteins and other solutes not absorbed by post-capillary venules, serving as a critical waste clearance mechanism for the CNS [19, 94, 95]. In vivo, imaging of fluorescently labeled albumin in animal models has shown a higher CSF clearance rate through the lymphatic system compared to the venous efflux pathway [96]. Through densely expressed aquaporin-4 (AQP4) water channels, the neuroglial barrier allows for the exchange of water and metabolites between perivascular CSF and ISF within the brain [21, 97]. Some studies indicate that meningeal lymphatic vessels are the primary pathway for CSF molecular efflux, and damage to these vessels may impede CSF clearance [98]. Neurons and glial cells have high metabolic rates and are highly sensitive to changes in the extracellular environment, suggesting a need for rapid clearance of ISF and solutes. In AQP4-deficient mouse models, clearance of CNS interstitial solutes and soluble amyloid-β was significantly reduced, highlighting the importance of this pathway for clearing toxic metabolites [19, 99]. Particularly noteworthy is evidence from animal and human studies suggesting that meningeal lymphatic function deteriorates with advancing age, and impaired meningeal lymphatic function exacerbates the pathological symptoms in animal models of Alzheimer's and Parkinson's diseases [86, 100-102]. Thus, dysfunction of the lymphatic system may lead not only to elevated ICP but also to impaired clearance of metabolic wastes from the CSF, thereby contributing to the symptoms of IIH.

The discovery of the lymphatic pathway has introduced new therapeutic avenues for IIH treatment. However, no strategies currently target this pathway to reduce ICP in IIH patients. Research indicates that metabolic dysfunctions, such as obesity, may contribute to IIH by modulating the lymphatic system. Nevertheless, the effects and precise pathological mechanisms remain unclear [25, 103].

## 3. Metabolic Abnormalities in IIH and Their Mechanisms Affecting IIH

**Figure 2.**
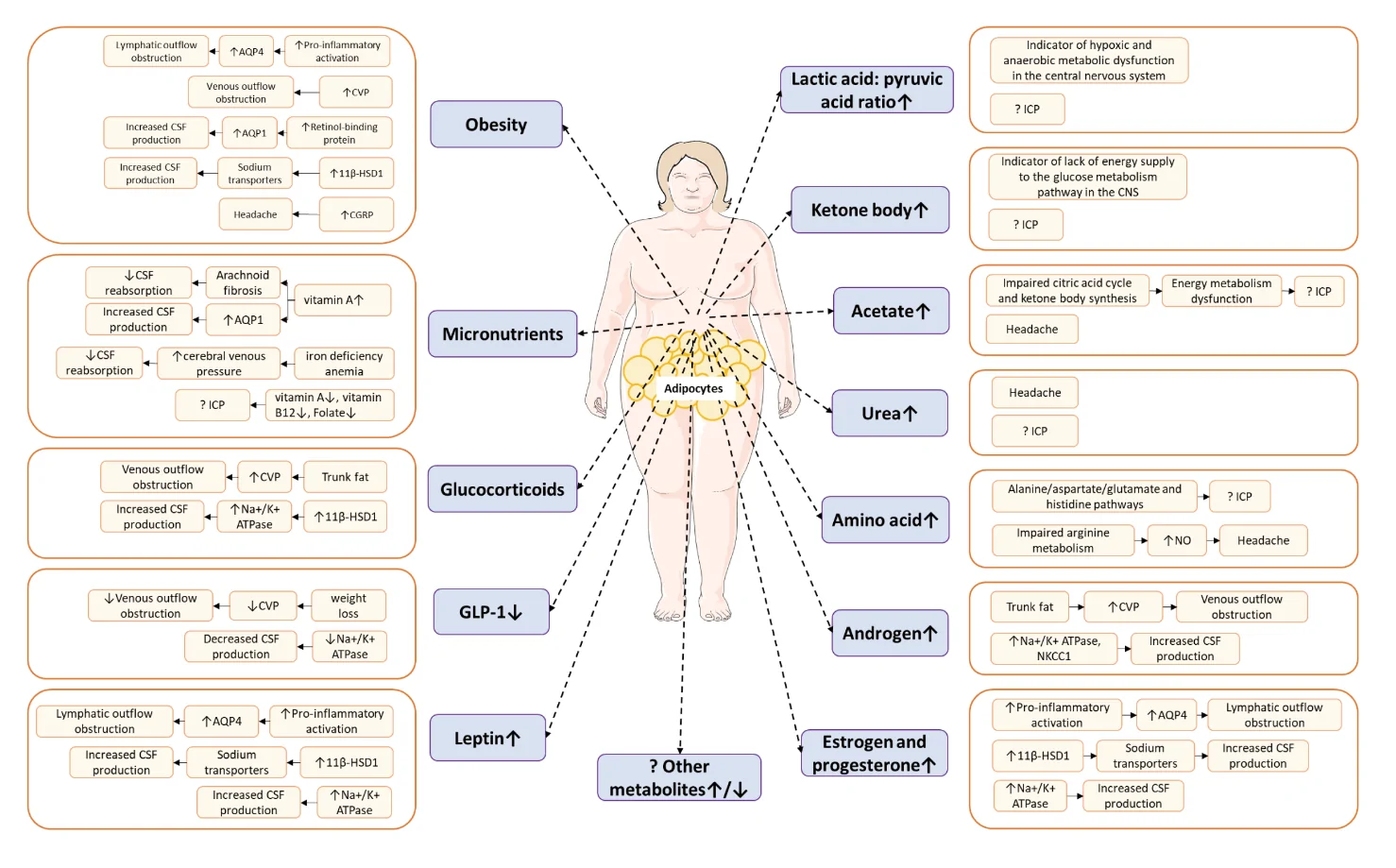
**Hypothesized mechanisms linking IIH with metabolic abnormalities**. This figure illustrates the potential links between IIH and various metabolic abnormalities, including obesity, micronutrients, glucocorticoids, GLP-1, leptin, estrogen, progesterone, androgens, amino acid metabolism, urea, acetate, ketone bodies, lactic acid: pyruvic acid ratio, etc. These factors above may contribute to typical intracranial hypertension and other symptoms observed in IIH. Abbreviations: IIH, idiopathic intracranial hypertension; CSF: Cerebrospinal fluid; AQP1, aquaporin 1; NKCC1, Na^+^-K^+^-2Cl^-^ cotransporter; ICP, intracranial pressure; CVP, central venous pressure; NO, nitric oxide.

Traditional theories regarding the pathogenesis of IIH have focused on excessive CSF production, impaired reabsorption, or circulatory dysfunction. However, the exact pathogenesis of IIH remains unclear, and no single molecular mechanism has been identified to explain its homeostatic disturbances. Increasing evidence suggests that IIH is not solely a CNS disorder but also involves systemic metabolic dysregulation (Fig. 2). Consequently, investigating the effects of various metabolic dysfunctions and hormonal imbalances on IIH is essential for gaining a deeper understanding of its pathological mechanisms and identifying potential diagnostic indicators and therapeutic targets.

### 3.1 The Most Common Chronic Metabolic Disorder in IIH-Obesity

Obesity represents the most prevalent risk factor for IIH [15]. The prevalence of IIH is on the rise in tandem with the escalating global obesity rate [3, 104]. Although a distinct association exists between obesity and IIH, this relationship is complex given the high prevalence of obesity in contrast to the rarity of IIH.

#### 3.1.1 Obesity and ICP

Obesity can increase intra-abdominal pressure, which subsequently elevates CVP and impairs cerebral venous outflow [18, 105, 106]. ICP elevation induced by a high-fat diet in experimental rats occurred along with a decreased capacity for CSF drainage [107]. It is also hypothesized that obesity can increase CVP by up to 20 mmHg, which in turn raises sagittal sinus pressure and obstructs CSF venous outflow pathways, ultimately leading to increased ICP [11]. Additionally, approximately 50%~60% of individuals with obesity develop obstructive sleep apnoea (OSA), and OSA frequently coexists with IIH [108, 109]. In patients with IIH, the combination of obesity and OSA can result in elevated cerebral venous pressure and ICP during nocturnal supine positioning, leading to epidural compression and CVSS, creating a positive feedback loop. Furthermore, some cases of obesity-related IIH have been successfully treated through bariatric surgery or weight loss alone [110, 111]. Weight gain represents a primary risk factor for IIH, whereas weight loss is associated with significant reductions in ICP levels, decreased papilledema, improved visual function, and reduced headache frequency [3, 104, 112, 113]. Consequently, actively treating underlying conditions associated with high ICP, such as obesity, may benefit IIH patients by reducing cerebral venous pressure.

#### 3.1.2 Obesity and Inflammation

Obesity is often associated with underlying metabolic disorders. The high prevalence of obesity among patients with IIH indicates that metabolic alterations could be a pathological mechanism underlying obesity-induced IIH. Additionally, IIH patients display a proclivity for accumulating adipose tissue in the trunk region, which is particularly associated with metabolic syndrome and insulin resistance [28, 114]. Women diagnosed with IIH have a significantly higher risk of developing gestational diabetes [115]. The altered metabolism and transcriptional characteristics of adipose tissue in IIH patients may predispose them to adipogenesis, fat accumulation, and excessive trunk fat [28]. Adipose tissue functions as an endocrine organ, secreting factors such as the pro-inflammatory cytokines, chemokines, and adipokines [52, 116].

There has been evidence to suggest that pro-inflammatory activation may contribute to the onset of IIH [117-120]. Pro-inflammatory markers, such as C-C motif chemokine ligand 2 (CCL2), are elevated in obese IIH patients [18, 25, 121]. Inflammatory mediators released by adipose tissue and astrocytes recruit leukocytes in the vicinity of blood vessels within the CNS, thereby increasing BBB permeability [25]. Additionally, elevated levels of AQP4 in astrocytic endfeet during inflammation may result in increased drainage of CSF through the lymphatic system, potentially leading to lymphatic congestion or overflow [25]. Moreover, hormonal dysregulation resulting from obesity may also contribute to IIH pathogenesis. The chronic inflammation and macrophage infiltration into peripheral fat cells promote the increased 11β-HSD1 activity, leading to cortisol release in the subcutaneous adipose tissue [122]. Studies have reported increased 11β-HSD1 activity in both obese individuals and those with IIH [123]. Additionally, reduced 11β-HSD1 levels have been linked with weight loss and decreased ICP in IIH patients [124]. Prolonged elevated cortisol levels have been demonstrated to enhance the secretion of pro-inflammatory mediators and may elevate CSF production by regulating sodium transporters in the choroid plexus [123, 125]. Moreover, adipose tissue releases retinol-binding protein and its metabolite retinoic acid has been demonstrated to promote CSF production by upregulating AQP1 expression in the choroid plexus [18, 121, 126]. The increased inflammation observed around blood vessels, in conjunction with the elevated CSF production, contributes to an accumulation of CSF in the PVS. This, in turn, causes lymphatic congestion and reflux obstruction [25].

A similar study using gadobutrol, a CSF tracer, showed reduced clearance rates in the PVS and interstitial spaces in IIH patients, particularly within the frontal and temporal lobes [127]. Interestingly, IIH incidence is usually higher in obese patients, who also bear a heavier burden of perivascular and lymphatic dysfunction, potentially supporting the hypothesis of lymphatic dysfunction in IIH. Although the pathological mechanisms behind this lymphatic dysfunction remain unclear, some studies suggest it may involve alterations at the neuroglial-vascular interface, particularly in astrocytic endfeet containing AQP4 channels. The high prevalence of obesity in women and its association with IIH have led to speculation about autoimmune, inflammatory, and metabolic causes of the dysfunction in IIH, possibly due to vascular inflammation or increased blood-brain barrier (BBB) permeability leading to AQP-4 loss. Thus, chronic inflammation and metabolic dysfunction in obese patients may contribute to the impaired lymphatic drainage pathway of CSF, and further evidence is needed to clarify this inference and explore the molecular mechanisms [25].

#### 3.1.3 Obesity and Headache

Disability in IIH is primarily caused by debilitating headaches that significantly impact the quality of life [128, 129]. Reducing ICP can improve headaches in IIH [111, 113], yet headache often persists despite normalization of ICP [128, 130-132]. Thus, there remains controversy regarding whether elevated ICP is the direct cause of the headaches in IIH.

##### 3.1.3.1 Mechanisms of Obesity and the Development of IIH Headache

Numerous basic science and clinical studies have verified the connection between obesity and calcitonin gene-related peptide (CGRP), a potent vasodilatory neuropeptide that also conveys injurious messages and comprises two variants: αCGRP and βCGRP. αCGRP and its receptors are extensively distributed in the CNS, especially in areas relevant to migraine pathophysiology, such as the trigeminal ganglion (TG) and the dura mater [133-138]. βCGRP, on the other hand, is predominantly found in the enteric nervous system. In a specific αCGRP knockout (αCGRP-/-) mouse model, these mice were prevented from diet-induced obesity and kept normal glycemic control, which suggests that CGRP assumes a significant part in metabolic regulation [139]. The effects of obesity on the trigeminal vascular system have been studied in rodents, showing diet-induced increases in basal CGRP release from meningeal afferent nerves in obese rats [140, 141]. Moreover, clinical studies have shown that obese women have elevated serum CGRP and amylin levels [50, 142, 143]. It has been proved that CGRP assumes a critical role in migraine attacks and serves as a critical neuropeptide for pain signaling [144]. Supporting this, clinical studies have reported elevated levels of CGRP in the saliva, serum, and CSF of migraine patients [145-148]. Moreover, exogenous CGRP injections induce a persistent migraine-like headache in patients [149]. Therefore, elevated CGRP levels in IIH patients may be one of the mechanisms of headache development, but further validation in IIH patients and models is still needed [150].

Amylin is a hormone conducive to satiety, and its analogs have been developed for the treatment of obesity [143, 151]. However, amylin is a well-characterized CGRP receptor agonist, sharing 25% to 50% sequence homology with CGRP. The utilization of its analogs for the management of obesity may potentially elevate the incidence of headaches in patients with IIH [152].

Increased cortical spreading suppression (CSD) has been observed in obese Zucker rats, indicating that obesity affects cortical excitability [153]. CSD is a depolarizing diffusion wave associated with reduced cortical activity and has been linked to migraine [154, 155]. Moreover, leptin levels are usually higher in obese patients. Notably, CNS leptin infusion in lean rats increases CSD, linking elevated serum leptin levels in migraine to increased migraine in obese females [153, 156, 157]. However, other physiologic changes caused by obesity may affect the quality of CSDs, warranting further investigation.

Growing evidence suggests that increased BMI, body fat percentage, and trunk obesity are associated with elevated ICP [158-162]. These studies indicate that factors contributing to headache occurrence are amplified by obesity. Although obesity is clearly associated with migraine and IIH, the underlying mechanisms require further clarification.

##### 3.1.3.2 Efficacy of CGRP-targeted Therapies in IIH

With the significant increase in IIH prevalence, the frequency of hospital visits due to headache and the associated financial burden have also risen [104, 163]. Therefore, headache treatment represents an urgent unmet need in IIH [13, 164].

There are few pharmacologic treatments for IIH, though new treatments are being developed to reduce ICP [165]. A 2015 Cochrane review on of IIH treatment concluded that, after a thorough evaluation of acetazolamide efficacy in IIH patients, evidence remains insufficient to recommend or reject its use for IIH treatment [16]. Nevertheless, acetazolamide is still considered the only available treatment for IIH [15]. Weight loss has been demonstrated to ameliorate headache in IIH, and thereby weight management is important in maintaining IIH remission [113, 166]. Despite the overwhelming evidence concerning the burden of IIH headache, treatments specifically targeting IIH headache management have not been adequately evaluated [128, 167]. Existing migraine prophylactic medications are employed off-label in routine care, with no evidence substantiating their effectiveness in the IIH population. In addition, these medications are typically poorly tolerated or contraindicated in IIH patients on account of their risk of intensifying obesity (a key factor driving elevated ICP) and mood disorders.

CGRP has been implicated in migraine etiology [146, 148, 168, 169]. Small-molecule receptor antagonists targeting CGRP signaling pathway or monoclonal antibodies against ligands or receptors have demonstrated clinical efficacy and are now licensed for migraine treatment [170-177]. A prospective study by Yiangou et al. demonstrated that erenumab significantly improves headache in IIH patients with resolving optic papilledema [178, 179]. As observed in other secondary headache disorders, elevated ICP may trigger CGRP release and trigeminal vascular activatio, persisting even after the initial damage from elevated ICP has subsided [180]. Thus, the CGRP may be a crucial regulator of headache in IIH, making CGRP targeting a promising therapeutic strategy for headache treatment.

### 3.2 Mechanisms of Metabolites' Influence on IIH

#### 3.2.1 Micronutrients

Individuals with obesity are more susceptible to developing IIH. Micronutrient deficiencies are prevalent among obese individuals, particularly deficiencies in vitamin A, vitamin B12, folate, and iron, with up to 85% of obese individuals presenting with at least one deficiency [48-51]. It can, therefore, be posited that a deficiency in micronutrients may be a contributing element to the development of IIH.

A number of micronutrients are closely associated with secondary intracranial hypertension, notably vitamin A and its derivatives [181]. Studies have demonstrated elevated concentrations of vitamin A derivatives in individuals diagnosed with IIH compared to controls [181]. Vitamin A can increase CSF production by upregulating AQP1 expression in the choroid plexus and may also elevate ICP by stimulating 11β-HSD1 activity. Although vitamin A overdose is an established risk factor for intracranial hypertension, evidence suggests that deficiency may also be a risk factor [182]. Patients with vitamin A deficiency have been shown to have increased ICP and symptoms such as headaches and visual impairment, and imaging findings of enlarged optic nerve sheaths [183]. The early application of vitamin supplements instead of carbonic anhydrase inhibitors has demonstrated rapid improvement [184]. However, the precise pathophysiological mechanisms linking vitamin A deficiency to intracranial hypertension remain to be elucidated. Animal experiments in rats and calves suggest that vitamin A deficiency may lead to arachnoid fibrosis, causing CSF absorption disorders and increased ICP [185]. This mechanism, however, requires further validation in humans.

Additionally, iron metabolism, specifically through iron deficiency anemia (IDA), has been linked to IIH. While anemia is often considered as a risk factor for IIH, the causal relationship remains speculative [186-188]. A recent systematic review showed that the incidence of anemia in IIH patients was 44% higher compared to the controls, with a comorbidity rate of 18.2% in IIH patients [189]. Another retrospective cohort study involving 607 IIH patients discovered an independent connection between anemia and IIH [190]. Furthermore, it has been reported that correcting anemia alone can completely alleviate the symptoms and signs of IIH [13, 191-194]. The exact etiology of increased ICP due to anemia remains unclear, though various mechanisms have been proposed, including (1) a relatively high-viscosity state leading to elevated cerebral venous pressure, reducing CSF absorption and ultimately increased ICP [192]; (2) tissue hypoxia altering cerebral hemodynamics, increasing BBB permeability and ICP [195]; and (3) iron-mediated hemostatic dysfunction affecting CSF dynamics and ICP [196]. The high-viscosity theory is generally considered the most plausible [197-200].

Inadequate intake or poor absorption are the primary causes of micronutrient deficiencies in specific populations. Given that obesity is a primary risk factor for IIH, current treatments often focus on calorie restriction over nutritional improvement. Consequently, calorie-restricted diets that lack adequate nutrition may worsen nutrient deficiencies and exacerbate the clinical manifestations of IIH [184]. Patients with intracranial hypertension of unknown etiology should be screened to exclude potential micronutrient-related risk factors for papilledema.

#### 3.2.2 Glucocorticoids

Glucocorticoids represent a category of pleiotropic steroid hormones that exert extensive influences on systemic metabolism and are implicated in the pathophysiology of IIH [81]. The bidirectional enzyme 11β-HSD mediates glucocorticoid action in target tissues and exists in two distinct isoforms. 11β-HSD1 primarily serves as a reductase, converting inactive glucocorticoids, such as cortisone, into their active form, cortisol, thereby promoting adipocyte differentiation and hepatic glucose output in adipose tissue [201, 202]. Conversely, 11β-HSD2 deactivates cortisol by transforming it into cortisone.

##### 3.2.2.1 11β-HSD1 and Adiposity

11β-HSD1 is expressed across various tissues, with hepatic 11β-HSD1 activity serving as the primary contributor to overall systemic 11β-HSD1 activity [203-207]. In obese patients, hepatic 11β-HSD1 is significantly reduced, while its activity in subcutaneous adipose tissue is increased [208, 209]. Despite the well-established link between obesity and IIH, recent data indicate that systemic metabolic disturbances in IIH are more severe than those associated with obesity alone [7, 28, 52]. The phenotype of increased adipose and systemic 11β-HSD1 activity in IIH patients exceeds that mediated solely by obesity [52]. Studies have reported elevated urinary cortisol and systemic 11β-HSD1 activity in IIH patients compared to obese controls [52]. Increased abdominal fat content in IIH patients may directly contribute to elevated systemic 11β-HSD1 activity as a result of the combined effect of enhanced activity across multiple tissues [28, 52]. Increased 11β-HSD1 activity in peripheral tissues leads to greater exposure to active glucocorticoids [30, 124, 210]. Adipose tissue in IIH patients has previously been observed to exhibit characteristics of glucocorticoid excess, including ribosomal subunit depletion, increased leptin secretion, and elevated lipid turnover [28]. Thus, IIH may represent a distinct 11β-HSD1 phenotype, separate from obesity, irrespective of whether systemic 11β-HSD1 changes occur without concomitant obesity.

##### 3.2.2.2 11β-HSD1 and Endocrine Pathways

Additionally, 11β-HSD1 can influence ICP by modulating endocrine and metabolic pathways. Research indicates that systemic dysregulation of 11β-HSD1 leads to increased central adiposity and elevated intra-abdominal pressure, resulting in increased CVP and upstream impairment of cerebral venous outflow [211, 212]. Moreover, 11β-HSD1 may directly affect ICP by modulating CSF production [52]. Key components of the 11β-HSD1 and glucocorticoid signaling pathways are expressed and functional in the choroid plexus and AGs, enhancing cerebral cortisol supply at these locations [124]. It is speculated that increased cortisol in the choroid plexus may bind to mineralocorticoid or intracellular glucocorticoid receptors, thereby upregulating the activity of the apical Na^+^/K^+^ ATPase, an essential transporter for CSF production [103]. Furthermore, in patients with IIH, reducing weight through therapeutic diets or bariatric surgery has been observed to reduce 11β-HSD1 activity and lower ICP, providing indirect proof for the pathophysiological function of the 11β-HSD1 pathway in IIH [213].

##### 3.2.2.3 11β-HSD1 and Therapy

Currently, 11β-HSD1 has been recognized as a promising therapeutic target for IIH, given its function in regulating endocrine and metabolic dysregulation. The specific inhibition of 11β-HSD1 (AZD4017) has been investigated in Phase II clinical trials for treating IIH [30, 214]. Results indicate that 11β-HSD1 inhibition in IIH patients has therapeutic effects, such as reducing ICP, improving lipid profiles (increasing high-density lipoprotein, improving the cholesterol/high-density lipoprotein ratio, and lowering cholesterol), reducing markers of liver dysfunction, and increasing lean muscle mass [215, 216]. Beyond directly inhibiting 11β-HSD1 activity and CSF production in the choroid plexus, AZD4017 treatment may also improve IIH risk factors, including obesity and lipid metabolism, thereby offering additional therapeutic benefits [52, 113, 217]. Moreover, elevated cortisol levels, known to drive cognitive dysfunction, have been documented in IIH patients and are associated with higher serum cortisol levels [218]. Studies have also indicated that the short-term memory of rodents can be enhanced by the inhibition of 11β-HSD1 activity [219]. Weight loss-induced normalization of serum cortisol levels may alleviate cognitive dysfunction in IIH patients, providing insights into the etiology of certain IIH symptoms [218, 220].

Many historical case reports have associated IIH with glucocorticoid therapy, despite glucocorticoids also being used to treat IIH [221]. Thus, the relationship between congenital glucocorticoids and IIH remains ambiguous, though 11β-HSD1 may be involved [103]. The regulation of 11β-HSD1 activity is species-, gender-, and tissue-specific. Notably, glucocorticoids themselves are capable of regulating 11β-HSD1 activity [222]. Studies have shown that cortisol, dexamethasone, and prednisolone can activate or inhibit 11β-HSD1 activity, contingent on the specific tissue under examination [223, 224]. Nevertheless, it remains unclear whether 11β-HSD1 expression across various tissues influences IIH, or if so, what the specific mechanism of this effect might be.

#### 3.2.3 GLP-1

GLP-1 is a peptide hormone mainly generated by intestinal L cells. Although a direct pathophysiological link between GLP-1 dysfunction and IIH remains unestablished, the therapeutic effects targeting GLP-1 have been confirmed [225].

GLP-1 promotes weight loss by regulating blood glucose, either by inhibiting glucagon secretion or by stimulating glucose-induced insulin release. Additionally, GLP-1 suppresses appetite by acting on receptors in areas like the hypothalamus, nucleus ambiguous, and ventral tegmental area [226-231]. Subsequent weight loss can facilitate CSF outflow pathways by reducing brain venous pressure, thereby lowering ICP. Moreover, GLP-1 inhibits renal diuresis by suppressing the Na^+^/H^+^ exchanger in the proximal renal tubule. Rodent models have demonstrated that GLP-1 receptors are present in the choroid plexus, hinting at a potential regulatory function in CSF production through a comparable mechanism of sodium ion transport [232-234]. Recent studies have shown rapid and sustained decreases in ICP and improvement in clinical symptoms in IIH patients treated with the GLP-1 receptor agonist exendin-4, even without significant weight loss [32]. The acute intervention with exendin-4 demonstrated a reduction in Na^+^/K^+^ ATPase activity in cell cultures, which serves as a pivotal regulator of CSF secretion [31]. These observations validate the hypothesis that GLP-1 exerts therapeutic effects in IIH by inhibiting the Na^+^/K^+^ ATPase transporter, thereby reducing CSF production [31, 32]. The circulating levels of GLP-1 have been demonstrated to increase significantly after weight loss surgery, particularly in procedures like Roux-en-Y gastric bypass (RYGB). Therefore, the therapeutic effects of weight loss surgeries in treating IIH may not solely result from weight loss [235]. Therefore, GLP-1 presents a promising therapeutic target for IIH.

#### 3.2.4 Leptin

Leptin is a peptide hormone secreted by adipose tissue, primarily targeting the hypothalamus to suppress appetite, enhance energy expenditure, and promote weight loss. Beyond the CNS, leptin enhances glucose uptake and utilization in peripheral tissues and organs, promotes lipolysis and fatty acid oxidation, and facilitates immune cell development and inflammatory factor production.

Leptin may contribute to IIH pathogenesis through mechanisms involving weight gain and pro-inflammatory effects. Firstly, it has been observed that serum leptin levels are elevated in IIH patients, exceeding those of obese controls, indicating systemic metabolic dysregulation and an increased risk of obesity [103]. Furthermore, the effect of leptin on adipose tissue inflammation depends on its plasma concentration; high leptin concentrations induce pro-inflammatory effects, while lower concentrations may have anti-inflammatory effects. Elevated leptin levels are known to activate recruited and resident macrophages in adipose tissue, inducing the production of inflammatory cytokines such as interleukin-12 (IL-12), interleukin-6 (IL-6), and tumor necrosis factor-alpha (TNF-α), thus exerting pro-inflammatory effects. Conversely, in leptin-deficient mice, even when subjected to a high-fat diet, macrophage infiltration and mRNA levels of inflammation markers, such as monocyte chemoattractant protein-1 (MCP-1) and TNF-α, in white adipose tissue are significantly reduced, suggesting that lowering leptin levels in high-fat conditions may alleviate adipose tissue inflammation. Regarding the mechanism of serum leptin's impact on ICP, it is speculated that elevated serum leptin levels in IIH patients may promote the release of pro-inflammatory factors, recruiting immune cells to the PVS of the CNS, causing perivascular inflammation, increased BBB permeability, and impaired CSF lymphatic drainage [25]. However, some reports have suggested that during inflammatory responses, AQP4 expression increases at the astrocytes endfeet, potentially facilitating lymphatic-mediated CSF flow as a compensatory mechanism, although insufficient to normalize ICP levels [236, 237]. Additionally, chronic inflammation and macrophage infiltration in peripheral adipocytes enhance the activity of 11β-HSD1, leading to increased cortisol release. Persistently elevated cortisol levels enhance pro-inflammatory mediator secretion and may increase CSF production by affecting sodium ion transporters in the choroid plexus [123, 125]. Consequently, further investigation is required to clarify the mechanisms by which serum leptin influences ICP.

Furthermore, current evidence regarding CSF leptin levels in IIH remains contradictory. Initially, CSF leptin levels were thought to increase in IIH patients; however, after adjusting for age and BMI, this association was found to be insignificant [119, 238, 239]. It is speculated that prolonged elevation of CSF leptin may induce excessive CSF production by increasing the activity of Na^+^/K^+^ -ATPases in the choroid plexus, similar to mechanisms observed in the kidneys [103]. Nevertheless, the aforementioned studies are constrained by factors such as fasting status, the selection of the control group, and small sample size, thereby being unable to establish a substantial correlation between CSF dysregulation and CSF leptin levels [103].

#### 3.2.5 Estrogen and progestin

The incidence of IIH shows a significant gender difference, with a notably higher prevalence in females than in males, suggesting a potential role of sex hormones in its pathogenesis. Studies have identified sex hormone receptors in the choroid plexus, indicating a regulatory role for sex hormones in CSF production [187]. Among these sex hormones, the involvement of androgens in the pathogenesis of IIH has been extensively studied, whereas research on estrogen and progestin is limited [52]. Some studies have reported elevated CSF estrogen levels in IIH patients, although these findings have not been confirmed in subsequent research [186, 240, 241]. Furthermore, the low sensitivity of radioimmunoassay in detecting low levels of hormones has posed a challenge to investigations into sex hormones in the pathogenesis of IIH [52].

Pregnancy is generally considered a significant risk factor for IIH due to physiological changes thought to increase ICP. These changes include heightened estrogen levels, weight gain, increased intra-abdominal pressure, and hypercoagulability-induced fibrinolysis, all potentially contributing to IIH by disrupting CVP and CSF reabsorption through thrombotic obstruction. It has been demonstrated that IIH can manifest at any stage of pregnancy but is more prevalent during the initial half of gestation [53]. Nonetheless, the incidence of IIH in pregnant women is similar to that of the general population, and subsequent pregnancies do not appear to elevate the risk of onset or recurrence [53]. In the absence of conclusive evidence for a causal link, IIH during pregnancy may occur through similar physiological mechanisms as in non-pregnant individuals, with prior associations possibly due to demographic overlap. Therefore, considering that IIH predominantly affects women of childbearing age, enhancing understanding of the complex interactions between sex hormones and ICP regulation is of great significance for guiding the management of women during pregnancy and for preventing IIH [242].

A possible connection between IIH and oral contraceptive use in women has been proposed. However, there is insufficient high-quality evidence to establish a direct link between modern oral contraceptives and IIH. Any observed association is likely incidental. Nonetheless, oral contraceptive use often leads to weight gain, which may predispose women to IIH [15]. Therefore, no definitive consensus exists on whether oral contraceptives increase the risk of IIH.

#### 3.2.6 Androgen

Research has shown that women with IIH exhibit distinctive characteristics related to circulating androgen excess, which suggests a potential involvement of androgens in the aetiology of IIH [54]. Similarly, IIH is primarily observed in obese women of reproductive age, a phenomenon that bears resemblance to the prevalence of polycystic ovary syndrome (PCOS) in this demographic. However, the pattern of androgen excess in women with IIH differs notably from that observed in individuals with PCOS or simple obesity. Specifically, there are significantly higher levels of serum testosterone, CSF dihydrotestosterone, and testosterone levels [27, 54, 186]. Additionally, studies have highlighted sex differences in the effects of androgens on human metabolism. While women are at risk of IIH due to androgen excess, men may be susceptible due to relative androgen deficiency [27, 52, 243-245].

Excess androgens in IIH may be attributed to increased activity of 5α-reductase. 5α-reductase is a key enzyme in cortisol metabolism and the conversion of testosterone to the potent dihydrotestosterone. It also exhibits sexual dimorphism in rodents, with higher levels in females. Studies have identified increased systemic activity of 5α-reductase in IIH patients, potentially reflecting a compensatory mechanism for excessive cortisol metabolism in IIH [50]. Additionally, 5α-reductase may contribute to elevated ICP in IIH by converting testosterone to dihydrotestosterone [50].

The mechanisms by which excess androgens cause CSF dysfunction to remain unclear. The human choroid plexus expresses androgen receptors and fundamental steroidogenic mechanisms activated by androgens [27]. However, it remains uncertain whether androgens are directly activated in the choroid plexus or in peripheral tissues, such as adipose tissue, before reaching the choroid plexus. Studies have shown that testosterone markedly increases the activity of Na^+^/K^+^-ATPases, which serve as a surrogate marker for CSF production, in rat choroid plexus cell lines [27]. Moreover, heightened activity of the choroid plexus transporter NKCC1 was observed in rats treated with testosterone, proving that testosterone may regulate CSF production via the choroid plexus [246]. Thus, it is hypothesized that excess androgens may induce excessive CSF production by directly stimulating Na^+^/K^+^-ATPase pump activity in the choroid plexus and increasing NKCC1 transporter activity. Furthermore, due to the lipogenic effects of androgens, IIH may also be associated with increased intra-abdominal pressure from abdominal fat accumulation, which may elevate jugular venous pressure and ICP [27]. To date, these findings above suggest that excess testosterone may be a potential pathogenic factor in IIH, and that targeted testosterone therapy could offer new therapeutic perspectives for IIH.

#### 3.2.7 Amino acid metabolism

Aberrant amino acid metabolism may offer new insights into the pathogenesis of IIH. Pathway enrichment analysis has pinpointed several serum amino acid metabolism pathways associated with clinical manifestations of IIH, such as the alanine/aspartate/glutamate and histidine pathways. However, the mechanism by which these pathways impact IIH remains unclear. Furthermore, studies have shown that disrupted arginine metabolism may be associated with the severe headache phenotype of IIH [129]. Nitric oxide (NO), synthesized from arginine by endothelial NO synthase, plays a crucial role in smooth muscle relaxation, vasodilation, and increased blood flow, making it a potent headache inducer [247-250]. Additionally, the involvement of arginine metabolism in NO synthesis has also been implicated in the pathogenesis and therapeutic response of migraines [249, 251, 252]. Moreover, impaired arginine metabolism may also result from oxidative stress and inflammation induced by obesity, which is a significant risk factor for IIH [253, 254]. Thus, disrupted amino acid metabolism, including arginine metabolism, has been identified as a potential factor in the clinical manifestations of IIH. However, the causal relationship between these amino acid metabolic processes and IIH remains uncertain due to numerous confounding factors in observational studies.

#### 3.2.8 Urea

Urea, a highly osmolar metabolite, has been shown to be associated with IIH and headache severity. Urea concentration differs between CSF and serum levels in IIH. Under physiological conditions, the concentration of urea in the CSF is slightly lower than that in the serum [255]. Additionally, baseline CSF urea levels decrease in patients with IIH. Furthermore, CSF urea levels and the CSF-to-serum urea ratio return to normal as symptoms resolve and ICP decreases [29]. Some studies suggest that the relative decrease in CSF urea concentration compared to serum urea concentration in IIH patients may present a compensatory mechanism, enhancing the osmotic gradient and promoting CSF outflow. Interestingly, a positive association has been observed between headache severity and CSF urea [29]. Thus, changes in the CSF, such as serum urea gradient in IIH patients, may significantly contribute to headaches. Moreover, the osmotic properties of urea can be exploited for treatment. Intravenous urea injection is widely used to reduce brain swelling in various conditions. It significantly reduces ICP in patients with acute brain injury, particularly in those with intracranial hypertension [256, 257]. Consequently, it can be reasonably proposed that CSF and serum urea may serve as potential targets for IIH treatment and efficacy assessment.

#### 3.2.9 Acetate

Altered CSF acetate levels in IIH patients correlate with the presence and characteristics of headaches. Baseline CSF acetate levels are significantly elevated in IIH patients and positively correlated with headache severity [29].

Acetate is a metabolite that can be converted to acetyl-CoA, which participates in several metabolic reactions, including the citric acid cycle and ketone bodies synthesis. These metabolic pathways supply energy to cells and play a crucial role in regulating the metabolism of nutrients such as carbohydrates, fats, and proteins, thereby contributing to maintaining normal physiological functions in the body. Moreover, leptin may regulate the expression of acetate and its metabolically related protein acetyl-CoA synthetase in IIH. Leptin resistance has been confirmed in IIH, and it has been reported that the expression of acetyl-CoA synthetase is suppressed in the absence of leptin [28]. This underscores acetate’s potential role in the metabolic dysregulation of IIH, supporting the hypothesis of IIH as a systemic metabolic disorder.

Furthermore, acetate may contribute to headache symptoms in IIH patients, either directly or indirectly through metabolites. While headache symptoms in IIH patients are often attributed to intracranial hypertension, not all patients experience complete relief from headache symptoms with intracranial hypertensive agents [128, 131, 132, 167]. Therefore, it is vital to investigate the metabolic pathological mechanisms of headache in IIH. On the one hand, acetate is known to be a major metabolite, leading to symptoms such as "hangover headache." In addition, in rodent models of migraine, acetate ingestion has been shown to enhance sensitivity to headache pathways via the trigeminal system [258]. The use of acetate as a renal buffering agent in dialysis can also lead to headaches [259]. Therefore, excessive acetate in IIH may directly exacerbate headache symptoms [29]. On the other hand, conversion of acetate to acetyl-CoA by acetyl-CoA synthetase can produce adenosine and adenosine monophosphate (AMP). Adenosine can stimulate pain nerve endings via adenosine A2A receptors and transmit pain by releasing histamine and other pain mediators from mast cells [260, 261]. Since reducing ICP is the primary treatment for IIH headache, reducing acetate levels may provide a new therapeutic approach for alleviating IIH headache symptoms.

#### 3.2.10 Ketone Bodies

Ketone body levels have been identified as potential diagnostic indicators for IIH. Research has found that baseline concentrations of the ketone bodies acetoacetate in the CSF of IIH patients are significantly higher [29].

Ketone bodies, including acetone, β-hydroxybutyrate, and acetoacetate, are intermediates of fatty acid oxidation in the liver, with typically lower levels under normal physiological conditions. Due to the presence of the BBB, only glucose and ketone bodies can enter the brain to provide energy. When the glucose supply is insufficient, ketone bodies can provide 25%~75% of the brain's energy. Previous research has indicated that individuals with IIH exhibit insulin insensitivity, which may lead to increased production of acetoacetate [28]. Decreased CSF levels of 3-hydroxybutyrate and acetone suggest inhibition of acetoacetate conversion to these ketone bodies or their utilization as alternative energy sources [262-264].

Additionally, altered levels of ketone metabolites return to normal after therapeutic weight loss in IIH patients [265]. Therefore, elevated CSF ketone bodies in IIH patients may reflect a compensatory response to the lack of energy supply in the glucose metabolism pathways of the CNS.

#### 3.2.11 Lactate: pyruvate Ratio

The lactate: pyruvate ratio has been demonstrated to be associated with elevated ICP in IIH, suggesting its potential as a diagnostic and prognostic indicator. Quantitative metabolomic profiling of IIH patients identified an imbalance in the lactate-to-pyruvate ratio and alterations in ketone body metabolism [29]. First, IIH patients had elevated baseline CSF and serum lactate: pyruvate ratios compared to controls, which correlated positively with elevated ICP. Similarly, elevated CSF lactate-to-pyruvate ratios were characteristics of elevated ICP in several conditions, including hydrocephalus, subarachnoid hemorrhage, and traumatic brain injury [266-269]. In addition, decreased levels of fumarate, an intermediate in the citric acid cycle, were found in the CSF of IIH patients, which may further elucidate the intracranial respiratory metabolic imbalance in IIH [269]. Thus, changes in CSF and serum lactate-to-pyruvate ratios may reflect anaerobic metabolism and mitochondrial energy metabolism dysfunction in IIH [270, 271]. Moreover, hypoxia and hypercapnia may lead to cerebral vasodilation and increase cerebral blood flow, potentially raising ICP [272]. Hypoxia-induced cerebral edema may also contribute to increased ICP through respiratory pause stress responses, excitatory neurotransmitter release, or BBB disruption [273-275]. Therefore, hypoxia and anaerobic metabolic disorders may play a role in elevated ICP in IIH, and enhancing oxygen supply may provide a new therapeutic approach to improve the prognosis in IIH. And the lactate-to-pyruvate ratio may serve as an indicator of mitochondrial energy metabolism and anaerobic metabolism dysfunction in IIH, offering potential diagnostic and prognostic value.

#### 3.2.12 Methylmalonic Acid

Furthermore, changes in methylmalonic acid levels may indicate ICP fluctuations in idiopathic IIH. The relative concentration of methylmalonic acid increased in the serum of IIH patients while decreasing in the CSF [29]. Methylmalonic acid is produced by microbial metabolism in the gut and can cross the BBB to reach the CNS [276, 277]. It is hypothesized that under pathological conditions, as ICP increases, the transport of methylmalonic acid across the BBB may decrease. With subsequent therapeutic intervention and ICP reduction, methylmalonic acid transport across the BBB may normalize, restoring its levels in both CSF and serum. Recent studies have highlighted the critical role of the gut microbiota in the pathogenesis of IIH [278]. Therefore, elevated serum methylmalonic acid may relate to gut microbiota changes. However, further research is needed to investigate whether and how the gut microbiota regulates IIH by modulating serum methylmalonic acid levels.

#### 3.2.13 Relationship between Specific Metabolites and Clinical Features

A study by Grech O et al. reported that several metabolites were expressed at abnormal levels in IIH and were associated with symptoms, though their relationship to the pathophysiological mechanisms of IIH remains unclear [29]. In this study, ICP measured by LP correlated with urea in urine, 2-hydroxyisobutyrate in serum, and galactitol in CSF. A significant decrease in ICP was linked to changes in urinary n-phenylacetylglycine, o-phosphocholine and CSF butanone. Papilledema measurements by retinal nerve fiber layer (RNFL) and optical coherence tomography (OCT) assessment in IIH patients correlated with serum isobutyric acid, o-acetylcarnitine, and urinary 2-hydroxyisobutyric acid. Baseline visual function measures were correlated with CSF threonate, lactate and fumarate, urine citrate, and serum 5-hydroxyindole-3-acetate. A correlation was identified between alterations in mean deviation of circumference and fluctuations in CSF leucine, along with alterations in serum 3-methyl-2-oxopentate, trimethylamine, valine, and urea. The severity of headaches was found to be correlated with the levels of acetate, urea, and creatine phosphate in CSF. The number of days per month suffering from headaches was also notably related to urinary creatine phosphate. During follow-up, alterations in the HIT-6 score were linked to changes in CSF butanone and urine urea. A significant association was observed between alterations in headache severity and changes in several urinary metabolites, including creatine phosphate, 2-hydroxyisobutyrate, and butanone [29].

Obesity is a classic feature of IIH. The study revealed a noteworthy relationship between BMI and several CSF metabolites, including 3-methyldioxyvalerate, isoleucine, methyl succinate, propylene glycol and threonine, and urinary 3-indolyl sulfate, n-acetylornithine, and n-phenylacetylglycine [29]. After the weight loss intervention, a pronounced correlation was observed between changes in BMI and CSF methyl ethyl keytone and, importantly, urinary urea, 3-indolyl sulphonic acid, and N-phenylacetylglycine [29]. The metabolites associated with ICP or BMI further supported the idea that IIH is a systemic metabolic disorder. In addition, a study by Kassubek R et al. found that melatonin levels were significantly elevated in IIH patients [114]. However, the mechanisms underlying interactions between the above metabolites and IIH require further investigation and validation.

## Conclusion

Despite recent advances in diagnosing and treating IIH, a comprehensive insight into its underlying pathophysiology remains elusive. Elevated ICP is a crucial component of IIH and a target of current treatments. However, the driving mechanisms of this process remain elusive and are not explicitly linked to this particular population. With a deeper understanding of IIH, it is increasingly recognized as not solely a CNS disorder but as having systemic metabolic features, particularly metabolic disturbances associated with obesity. Thus, the etiology of IIH may involve systemic metabolic dysregulation that subsequently affects CSF production and circulation, ultimately leading to increased ICP due to CSF imbalance. Although previous research has provided insight into metabolic factors and CSF balance mechanisms potentially contributing to the IIH pathogenesis, findings remain mostly observational and controversial. Further research is necessary to elucidate and validate the pathological mechanisms through which metabolic markers contribute to IIH, offering new diagnostic markers and therapeutic targets for clinical management.
